# Temperature-Automated Calibration Methods for a Large-Area Blackbody Radiation Source

**DOI:** 10.3390/s24051707

**Published:** 2024-03-06

**Authors:** Wenhang Yang, Chen Cao, Pujiang Huang, Jindong Bai, Bangjian Zhao, Shouzheng Zhu, Haijun Jin, Ke Jin, Xin He, Chunlai Li, Jianyu Wang, Shijie Liu, Hongxing Qi

**Affiliations:** 1 Hangzhou Institute for Advanced Study, UCAS, Hangzhou 310024, China; yangwenhang22@mails.ucas.ac.cn (W.Y.); caochen@ucas.ac.cn (C.C.); xinhe@ucas.ac.cn (X.H.);; 2 Shanghai Institute of Technical Physics, Chinese Academy of Sciences, Shanghai 200083, China; 3 University of Chinese Academy of Sciences, Beijing 100049, China

**Keywords:** infrared thermometer, large-area blackbody radiation source, auto-calibration

## Abstract

High-precision temperature control of large-area blackbodies has a pivotal role in temperature calibration and thermal imaging correction. Meanwhile, it is necessary to correct the temperature difference between the radiating (surface of use) and back surfaces (where the temperature sensor is installed) of the blackbody during the testing phase. Moreover, large-area blackbodies are usually composed of multiple temperature control channels, and manual correction in this scenario is error-prone and inefficient. At present, there is no method that can achieve temperature-automated calibration for a large-area blackbody radiation source. Therefore, this article is dedicated to achieving temperature-automated calibration for a large-area blackbody radiation source. First, utilizing two calibrated infrared thermometers, the optimal temperature measurement location was determined using a focusing algorithm. Then, a three-axis movement system was used to obtain the true temperature at the same measurement location on a large-area blackbody surface from different channels. This temperature was subtracted from the blackbody’s back surface. The temperature difference was calculated employing a weighted algorithm to derive the parameters for calibration. Finally, regarding experimental verification, the consistency error of the temperature measurement point was reduced by 85.4%, the temperature uniformity of the surface source was improved by 40.4%, and the average temperature measurement deviation decreased by 43.8%. In addition, this system demonstrated the characteristics of strong environmental adaptability that was able to perform temperature calibration under the working conditions of a blackbody surface temperature from 100 K to 573 K, which decreased the calibration time by 9.82 times.

## 1. Introduction

Infrared remote sensing is a principal method for observing Earth from space, and it is crucial for the research field of Earth science. Infrared remote sensing technology can accurately provide a wealth of information about the Earth’s surface or environmental conditions. Consequently, it is extensively utilized in military reconnaissance, meteorological observation, environmental monitoring, resource surveys, and disaster prevention, among other applications [1,2,3,4,5,6,7]. With the continuous development of infrared remote sensing technology, the measurement of infrared radiation characteristics has become an essential tool for acquiring features and identifying complex or weak targets [8,9,10,11]. Quantitative data acquisition from infrared cameras necessitates radiometric calibration, which correlates the grayscale values of a digital image produced by the camera with radiometric quantities, establishing a quantitative relationship between the input and output [12,13].

Blackbody radiation sources are a critical component in the calibration of infrared remote sensing systems. Infrared measurement devices require recalibration of the system before field measurements to update calibration data. For large-aperture radiometric measurement devices, it is necessary to prepare large-area blackbody sources that can cover the aperture [7,14]. The goal of developing large-area blackbody sources is to provide a highly uniform and precisely known radiative temperature when periodically observed using optical devices such as cameras. To meet this requirement, the blackbody itself must possess high reliability and uniformity.

However, the larger the blackbody radiation area is, the worse the temperature uniformity and accuracy are. Lani et al. [15] studied the temperature accuracy and uniformity of blackbody radiation sources. The results indicated that it is challenging to achieve a uniform temperature distribution on the surface of large blackbody sources, leading to deviations in temperature calibration and infrared device measurements. Therefore, the development of high-performance large-area blackbody radiation sources is of significant importance to meet the requirements of radiometric calibration for large-aperture infrared measurement devices.

The large surface source blackbody is composed of multi-channel temperature control to improve the temperature uniformity of the surface source. For example, the 2200 × 2200 mm blackbody surface source in our laboratory is composed of 64 temperature control channels. It divides the size of a 2200 × 2200 mm blackbody into 64 areas, 8 × 8 in total, and each area is controlled by a temperature control system to control the temperature of that area. Each temperature control system is called a temperature control channel of the blackbody. The consistency error of the temperature measurement point refers to the positional discrepancy of the temperature measurement points across different temperature control channels during the calibration process. During the temperature-correction procedure, the measurement point of each channel will have a certain distance error relative to the center of the blackbody, which directly affects the temperature uniformity correction of the blackbody source and, consequently, the calibration performance of the blackbody source. Most large-area blackbodies rely on manual measurement methods for testing and calibrating the errors of each temperature control channel, whereby an infrared thermometer is fixed on a tripod and the measurement point is changed by moving the tripod [16]. This method is not only inefficient, but also poses difficulties in operation, especially within extreme-temperature environments (with high-temperature blackbody sources reaching up to 1473 K and low-temperature sources down to 100 K), presenting certain risks to operators [17,18]. Moreover, the consistency of multi-channel temperature measurement points is challenging to ensure. To the best of the authors’ knowledge, there is currently no system in the field of large-area blackbody research that is capable of temperature auto-correction during the design and testing processes of large-area blackbodies. If temperature-automated calibration is achieved, the calibration efficiency of the blackbody during the testing phase will be higher, and the performance of the corrected blackbody will also be better. Because the temperature-automated calibration can achieve high-precision position control, the position of the temperature measurement points for each temperature measurement channel can be almost consistent, while manual correction has a significant error in the position of the temperature measurement points for each temperature measurement channel. The higher the consistency of temperature measurement points, the more accurate the calibrated correction value, resulting in smaller temperature differences between channels and better temperature uniformity of the blackbody radiation source.

This paper proposes a large-area blackbody temperature auto-correction system designed to automatically correct the discrepancy between the displayed temperature and the actual temperature during the design process of large-area blackbodies. A small-scale experimental system was constructed and validated under both high- and low-temperature conditions. The consistency errors of the temperature measurement points across all channels and the correction efficiency were compared between manual and automatic calibration. Furthermore, the maximum temperature accuracy error and the temperature uniformity of the same blackbody source were compared after the same number of corrections using both automatic and manual methods. These comparisons demonstrate the significant application value of this system.

## 2. Auto-Correction System Calibration Principle for Infrared Thermometers

### 2.1. Method for Calibrating the Temperature of the Blackbody

**Figure 1:**
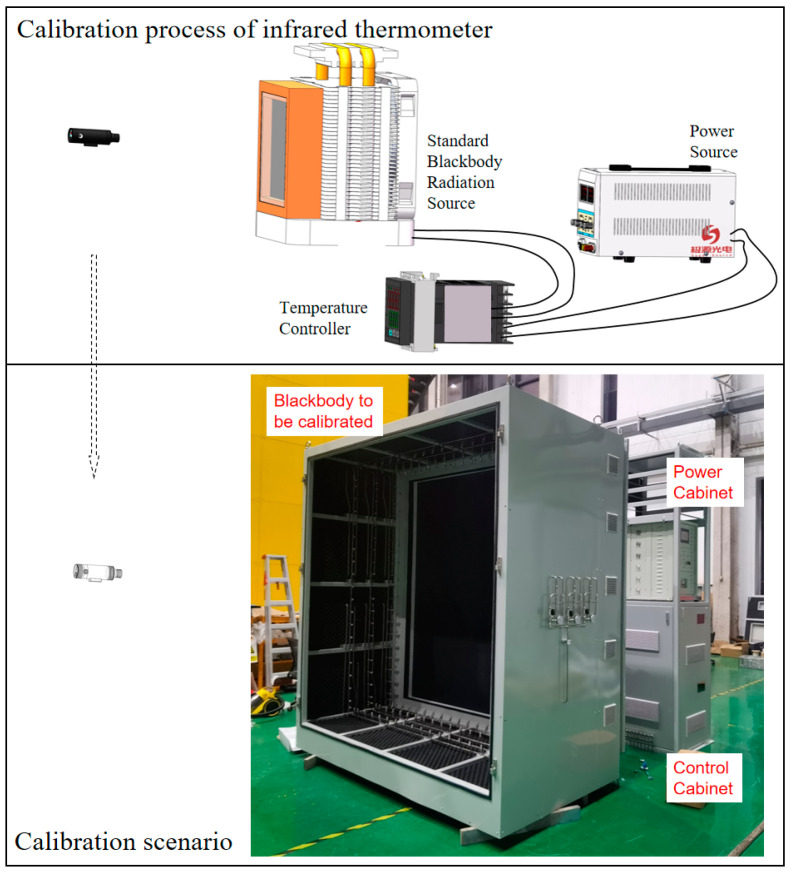
Calibration equipment and process.

During the design process of blackbody surface sources, a temperature discrepancy exists between the uncalibrated blackbody surface temperature and the standard temperature. This discrepancy primarily comprises the following components: (1) the blackbody heating and temperature measuring elements are positioned at the back of the blackbody surface, whereas calibration utilizes the front surface, leading to a temperature difference due to the thickness of the radiating surface; (2) the inherent temperature measurement errors of the temperature sensors; and (3) temperature drifts over time in the temperature sensing elements. As the blackbody serves as a standard reference, it is imperative to correct these errors. The measurement accuracy of IR cameras is usually ±2 °C or ±2%, and the greater value is valid (for the most accurate systems, ±1 °C or ±1%, ±5 °C or ±5% for low-end IR cameras) for pyrometers, ±1 °C or ±1% or ±2 °C or ±2% [19,20]. The challenge lies in calibrating a blackbody surface source with a precision index of 0.01 K. To achieve this objective, it is essential to clarify three concepts: accuracy, resolution, and display precision. The accuracy of a thermopile infrared sensor by our labrary is 0.1 K, while its resolution and display precision are 0.001 K. This precision refers to the accuracy of temperature measurement at discrete temperature points rather than across a continuous temperature range. For instance, the temperature measurement precision at commonly used blackbody temperatures such as 283 K and 303 K achieves a 0.1 K accuracy. Taking a blackbody standard of 303 K as an example, due to the accuracy limitations of infrared thermometers, different units of the same model may display a precision range from 302.900 K to 303.100 K, all potentially representing the standard 303 K. For a thermopile infrared sensor, functionality is predicated based on the thermoelectric effect, where it converts the received infrared radiation signal into an electrical signal. This is achieved by establishing a relationship between the electrical signal and the temperature value through the voltage difference across the hot and cold junctions (with the cold junction typically being at a standard temperature). This signifies that its deviation at a benchmark temperature of 303 K is a constant value within the ambit of 303 ± 0.1 K. This represents a steady-state error. In other words, for an individual infrared thermometer, the measurement error at the standard 303 K is a constant steady-state error, whereas for multiple infrared thermometers, the error at 303 K falls within a random error range of 303 ± 0.1 K, necessitating experimental verification of each infrared thermometer’s steady-state error at a single temperature point. The correction of steady-state errors is achieved through calibration. An infrared thermometer calibrated to 0.01 K precision corrects the steady-state error of the blackbody surface source, thus achieving temperature control at the 0.01 K level. The calibration method is as follows:

A comparative method was employed for calibration using a standard blackbody radiation source as the reference and a radiation thermometer as the comparator to calibrate the radiation temperature of the blackbody radiation source. The calibration equipment and process are shown in Figure 1. Firstly, we used a calibrated standard small blackbody to calibrate the thermopile infrared sensor. Then, we used the calibrated thermopile infrared sensor to calibrate the large-area blackbody radiation source. The low-temperature blackbody in the fourth part of the experiment was conducted in a vacuum tank, while the medium-temperature blackbody and high-temperature blackbody were conducted indoors with the blackbody compartment door closed.

The standard and the blackbody radiation source to be calibrated were stabilized at the same temperature. The radiation thermometer was used to measure the radiation temperature display values of both the standard and the blackbody radiation source to be calibrated. The radiation temperature of the blackbody radiation source to be calibrated was calculated using Equation (1):

(1) $$T_{c}=T_{s}+T_{cr}-T_{sr}=T_{s}+\Delta T_{r},$$

where $T_{c}$ is the radiation temperature of the blackbody radiation source to be calibrated, $T_{s}$ is the radiation temperature of the standard blackbody radiation source, $T_{sr}$ is the radiation thermometer’s measurement value of the standard blackbody radiation source’s radiation temperature, $T_{cr}$ is the radiation thermometer’s measurement value of the blackbody radiation source to be calibrated, and $\Delta T_{r}$ is the difference between $T_{cr}$ and $T_{sr}$.

The radiation temperature of the standard blackbody radiation source was calculated using Equation (2):

(2) $$\int_{\lambda_{1}}^{\lambda_{2}}L(\lambda ,T_{s})d\lambda =\int_{\lambda_{1}}^{\lambda_{2}}\varepsilon_{s}L(\lambda ,T_{t})d\lambda +\int_{\lambda_{1}}^{\lambda_{2}}\left(1-\varepsilon_{s})L(\lambda ,T_{am}\right)d\lambda ,$$

where $L(\lambda ,T_{t})$ is the spectral radiance of the blackbody, given by Planck’s law [21,22], in watts per square meter per steradian per micrometer $W/(m^{3}\cdot sr)$, $\lambda_{1}$, and $\lambda_{2}$ are the upper and lower limits of the radiation thermometer’s working waveband (μm), respectively; $T_{t}$ is the actual temperature measured by the reference thermometer of the standard blackbody radiation source, $T_{am}$ is the environmental temperature of the standard blackbody radiation source, and $\varepsilon_{s}$ is the effective emissivity of the standard blackbody radiation source.

### 2.2. Temperature Test Deviation

The calibration model for the radiation temperature of the blackbody radiation source, the radiation thermometer display value, and the difference between the two can be represented by Equation (3):

(3) $$T_{s}=T_{sr}+\Delta T_{1},$$

In the equation, $\Delta T_{1}$ represents the difference between $T_{s}$ and $T_{sr}$.

The relationship between the radiation temperature of the blackbody radiation source to be calibrated, the radiation thermometer display value, and the difference between the two can be represented by Equation (4):

(4) $$T_{c}=T_{cr}+\Delta T_{2},$$

In the equation, $\Delta T_{2}$ represents the difference between $T_{c}$ and $T_{cr}$.

**Figure 2:**
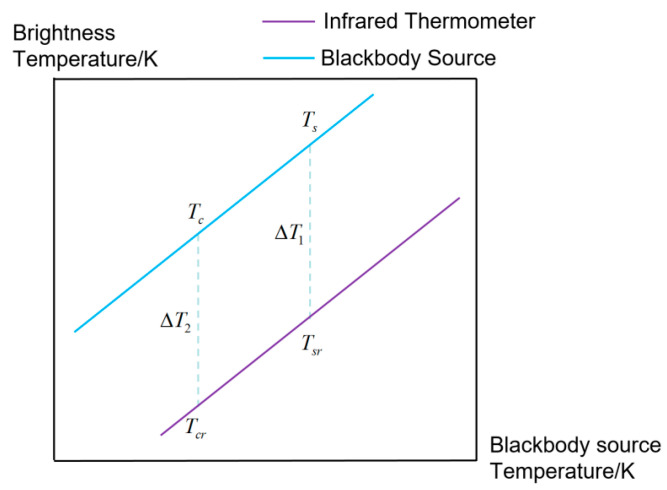
Relationship between blackbody radiation source radiation temperature and the thermometer display value.

The relationship between the radiation temperature of the blackbody radiation source and the thermometer display value is shown in Figure 2.

During calibration, the actual temperatures of the standard blackbody radiation source and the blackbody radiation source to be calibrated are the same, and the corresponding radiation temperatures of the two blackbody radiation sources are close. It can be assumed that the difference between the radiation thermometer display value and the blackbody radiation source radiation temperature is constant within a small range, $\Delta T_{1}\approx \Delta T_{2}$, leading to Equation (5):

(5) $$T_{cr}-T_{sr}\approx T_{c}-T_{s},$$

Equation (6) can be derived from Equation (5):

(6) $$T_{c}=T_{s}+\Delta T_{r},$$

The standard blackbody radiation source was aimed at, the position was adjusted, and the measurements were taken. The reference thermometer measurement values and radiation thermometer measurement values of the standard blackbody radiation source were recorded three times.

The blackbody radiation source to be calibrated was aimed at, the position adjusted, and measurements taken. The radiation thermometer measurement values were recorded three times. A total of three sets of comparative measurements were conducted for each calibration temperature point.

During each set of comparative measurements, the standard and the blackbody radiation source to be calibrated should be measured alternately at equal time intervals.

The radiation temperature of the standard blackbody radiation source $T_{s}$ and $\Delta T_{r}$ for each comparative measurement was calculated, using Equation (3). The average values of $T_{s}$ and $\Delta T_{r}$ from multiple comparative measurements were calculated using Equations (7) and (8):

(7) $$\bar{T_{s}}=\frac{\sum_{h=1}^{3}\sum_{k=1}^{3}T_{s\cdot hk}}{3\times 3},$$

In the formula, $\bar{T_{s}}$ denotes the mean brightness of the standard blackbody radiation source at the average temperature $T_{s}$, and $T_{s\cdot hk}$ represents the radiation temperature of the standard blackbody radiation source for the h-th group at the k-th measurement.

(8) $$\bar{\Delta T_{r}}=\frac{\sum_{h=1}^{3}\sum_{k=1}^{3}\Delta T_{r\cdot hk}}{3\times 3},$$

where $\Delta T_{r\cdot hk}$ represents the difference between $T_{cr}$ and $T_{sr}$ for the h-th group at the k-th measurement, while $\bar{\Delta T_{r}}$ is the average value of $\Delta T_{r\cdot hk}$.

The radiation temperature of the blackbody radiation source to be calibrated was calculated using Equation (9):

(9) $$T_{c}=\bar{T_{s}}+\bar{\Delta T_{r}},$$

The temperature deviation of the calibration blackbody was calculated according to Equation (10):

(10) $$T_{TA}=T_{c}-T_{a}$$

### 2.3. Uniformity of Temperature Test

The temperature points were selected and evenly distributed within the temperature range of the blackbody radiation source. The uniformity test positions were selected at the middle, upper left, lower left, lower right, and upper right of each channel of the blackbody radiation source. The temperature of the blackbody radiation source being calibrated was set at the test temperature point, with a temperature stability of no more than 0.1 K and 0.1%|t| of the larger one (t is the calibration point temperature value) within 10 min. The position of the radiation thermometer was adjusted to make it coaxial with the center of each channel of the blackbody radiation source; at this time, the radiation thermometer was aimed at the center position of each channel of the blackbody radiation source. A total of three measurements were performed at each position. The temperature uniformity of each channel is the difference between the temperature at each point and the center temperature, calculated according to Equation (11):

(11) $$\Delta T_{Fi}=\bar{T_{cri}}-\bar{T_{crc}},$$

In the formula, $\Delta T_{Fi}$ is the difference between the temperature at each point and the center temperature; $\bar{T_{cri}}$ is average the measurement of the radiation temperature of the upper, lower, left, and right parts of the blackbody radiation source (i = 1, 2, 3, 4); $\bar{T_{crc}}$ is the average radiation temperature at the center position of the blackbody radiation source.

The temperature uniformity of the blackbody surface source is the difference between the maximum and minimum values of all temperature measurement points in each channel, calculated according to Equation (12):

(12) $$\Delta T_{F}=\max(\bar{T_{cri\max}},\bar{T_{crc\max}})-\min(\bar{T_{cri\min}},\bar{T_{crc\min}}),$$

In the formula, $\Delta T_{F}$ is the temperature uniformity of the blackbody surface source, $\max(\bar{T_{cri\max}},\bar{T_{crc\max}})$ is the maximum temperature of all temperature measurement points in each channel, and $\min(\bar{T_{cri\min}},\bar{T_{crc\min}})$ is the minimum temperature of all temperature measurement points in each channel.

### 2.4. Auto-Correction System for the Focusing and Motor Control Method

The large-area blackbody temperature auto-correction system primarily consists of a three-coordinate positioning system and an infrared temperature measurement system. The three-coordinate positioning system is composed of a focus adjustment platform, a horizontal movement platform, and a lifting platform. The focus adjustment platform is used to adjust the focus distance of the infrared temperature measurement system, achieving Z-direction distance control. The horizontal movement and lifting platforms were utilized for X–Y plane positioning, enabling the temperature measurement of the same position across different temperature measurement channels. The infrared temperature measurement system comprises an insulation cover, an industrial camera, and two infrared thermometers (thermopile infrared sensors), which are employed to acquire the temperature data on the blackbody surface. The industrial camera captures continuous samples of the focused laser cross spot from the infrared thermometer and uses a focus detection algorithm to determine the optimal focus point, thus identifying the most accurate temperature measurement point for the infrared thermometer. The use of two infrared thermometers first provided timely warnings when the temperature drift of the calibrated dual infrared temperature sensors was too large, in which case the infrared thermometer had to be replaced or recalibrated. Second, in terms of checking the uniformity of the surface source temperature, the main existing method uses an infrared imager, with the NETD of current mid-to-high-end imagers typically at the level of 0.1 K, and the accuracy was roughly ±2 K. However, using calibrated dual infrared thermometers, the temperature uniformity of the surface source at the level of 0.01 K could be roughly estimated.

**Figure 3:**
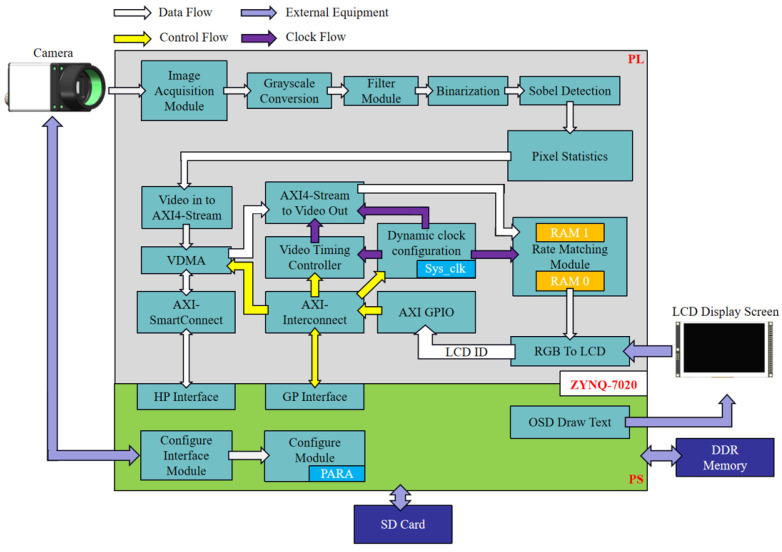
Focus detection algorithm flowchart.

The flowchart of the focus detection algorithm for the dual infrared thermometers is shown in Figure 3. Initially, the industrial camera is configured by the PS side, and after configuration, the captured image is processed in the PL part. The image is converted to grayscale, followed by filtering and binarization operations, and finally, the Sobel detection algorithm is applied to the binarized image for edge detection. The largest enclosed image area extracted is then subjected to pixel count detection. After the focusing algorithm, the number of focused laser, and red pixels is counted to determine the most accurate focus point. Once the determination is complete, the infrared thermometer performs temperature detection, and the detected temperature data are sent to the host computer.

The algorithm formula for RGB to the YCbCr color space conversion is as shown in Equation (13):

(13) $$\left\{\begin{array}{c} Y=0.299*R+0.587*G+0.114*B\\ Cb=-0.169*R-0.331*G+0.5*B+128\\ Cr=0.5*R-0.419*G-0.081*B+128 \end{array}\right.,$$

Since Verilog HDL cannot perform floating-point operations, the formula was converted by scaling up 256 times and then shifting right by 8 bits, (0.083 = 00010101), as shown in Equation (14):

(14) $$\left\{\begin{array}{c} Y=(77*R+150*G+29*B)>>8\\ Cb=(-43*R-85*G+128*B)>>8+128\\ Cr=(128*R-107*G-21*B)>>8+128 \end{array}\right.,$$

To prevent negative numbers during the calculation process, we further transformed the above formula to obtain Equation (15):

(15) $$\left\{\begin{array}{c} Y=(77*R+150*G+29*B)>>8\\ Cb=(-43*R-85*G+128*B+32768)>>8\\ Cr=(128*R-107*G-21*B+32768)>>8 \end{array}\right.,$$

The filtering module is responsible for the noise filtering of image data, eliminating Gaussian noise. Its formula is as follows:

(16) $$\begin{array}{c} g(x,y)=\{f(x-1,y-1)+f(x-1,y+1)+f(x+1,y-1)+f(x+1,y+1)+[f(x-1,y)\\ +f(x,y-1)+f(x+1,y)+f(x,y+1)]*2+f(x,y)*4\}/16, \end{array}$$

where f(x,y) is the grayscale value of the pixel point in the original image, and g(x,y) is the value after Gaussian filtering. The division by 16 in the formula facilitates implementation within the hardware.

**Figure 4:**
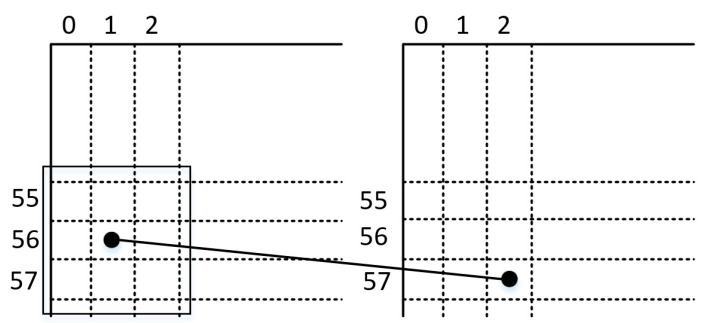
Gaussian filter pixel shift diagram.

The above formula can be structured into a 3 × 3 mask. As shown in Figure 4, the left side is the original image and the right side is the image output after Gaussian filtering. If Gaussian filtering is applied to the green point in the 56th row and 1st column on the left, the filtered output point will be located in the 57th row and 2nd column (the red point on the right). This means that after Gaussian filtering, the output image will move down one row and one column to the right.

**Figure 5:**
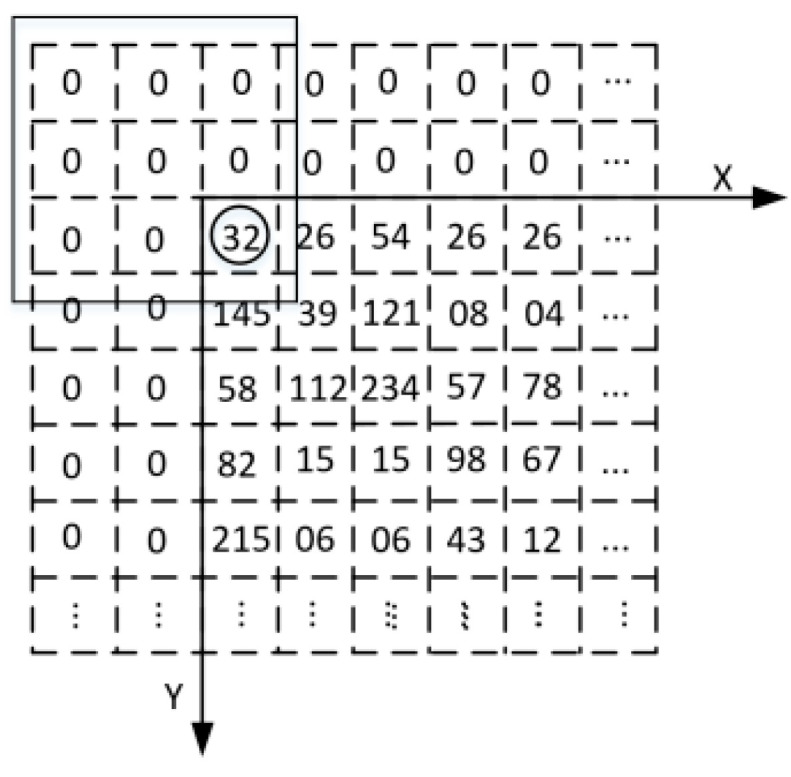
Gaussian filter boundary value processing.

As shown in Figure 5, the value of the original image at row 0, column 0 is 32 (indicated by the black circle in the figure). If Gaussian filtering is applied to this point, it is found that there are no values on its left and upper boundaries. A solution was proposed: add two rows of zeros on its upper boundary and two columns of zeros on its left boundary to form a 3 × 3 matrix. Gaussian filtering can then be performed using this matrix, and similar processing is applied to other edge points [23].

The Sobel operator is primarily used for edge detection. The correction environment for large-area blackbody radiation source is usually a closed indoor environment with almost no external interference, so the Sobel algorithm is used to achieve edge detection. Technically, it is a discrete differential operator that computes the approximate value of the gradient of the image brightness function. Applying this operator at any point in the image will produce a corresponding gradient vector or a normal vector [24].

The Sobel convolution factor consists of two sets of 3 × 3 matrices, one for the horizontal direction and the other for the vertical direction [25]. Convolution with the image plane yields approximate values of the brightness differences in the horizontal and vertical directions, respectively. If A represents the original image, and Gx and Gy represent the image grayscale values after horizontal and vertical edge detection, respectively, their formulas are as follows:

(17) $$G_{x}=\left[\begin{array}{ccc} -1 & 0 & +1\\ -2 & 0 & +2\\ -1 & 0 & +1 \end{array}\right]*A,$$

(18) $$G_{y}=\left[\begin{array}{ccc} +1 & +2 & +1\\ 0 & 0 & 0\\ -1 & -2 & -1 \end{array}\right]*A,$$

The grayscale value of each pixel in the image is combined using the following formula to calculate the magnitude of the grayscale at that point:

(19) $$G=\sqrt{G_{x}^{2}+G_{y}^{2}},$$

If the gradient G is greater than a certain threshold, the point (x, y) is considered an edge point.

**Figure 6:**
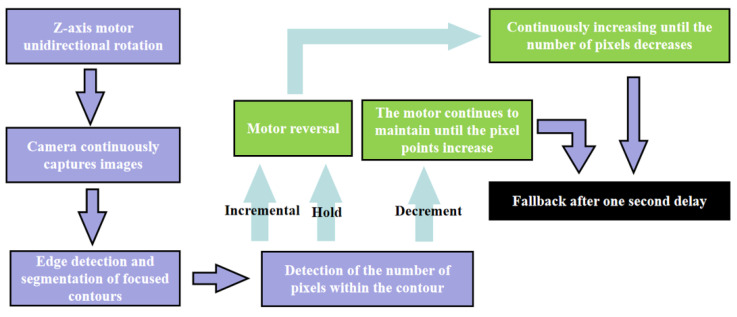
Algorithm for finding the optimal focus point of the adjustment platform.

The optimal focusing strategy for the Z-axis focusing adjustment platform is depicted in Figure 6. Initially, the Z-axis motor is set to move in a single direction (arbitrary), and the camera mounted on the Z-axis continuously captures the pattern of the cross-laser focus from the infrared thermometer. The image undergoes pixel point collection and state determination based on the procedure outlined in Figure 3, assessing changes in the number of pixel points: if the count is increasing or remains constant, the motor reverses its direction after a one-second delay following the increase in pixel points; if the count is decreasing, the motor continues its current motion until the pixel points increase, followed by a one-second delay before retreating. The process concludes once the optimal focus position for the motor is ascertained. Upon determining the optimal focus position of the motor, the infrared thermometer acquires the corresponding temperature data and subsequently transmits this information to the host computer.

## 3. Simulation of Focusing Procedure and Temperature Correction

**Figure 7:**
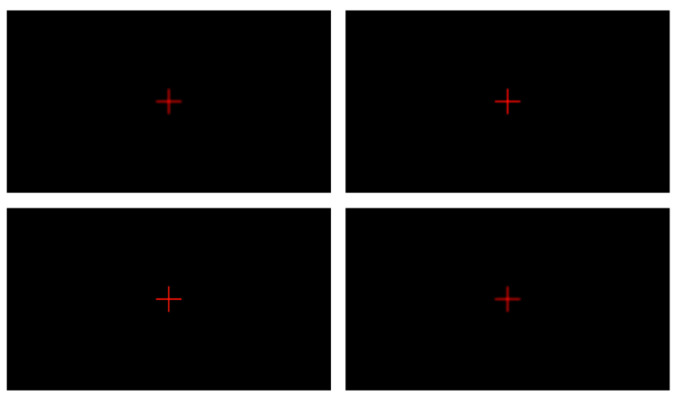
Sampling of changes in blur of focusing cross laser.

**Figure 8:**
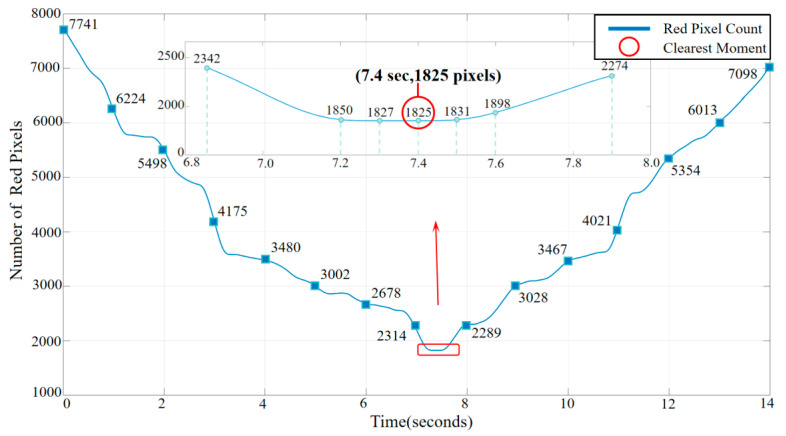
Red pixel count over time.

Using Matlab 2016a, a video was generated featuring a red cross on a pure black background, with the blurriness of the cross varying from high to low, and then increasing again over a duration of 14 s at 60 frames per second. At the 7.4 s mark, the blurriness reached its minimum, as depicted in Figure 7. The video was subjected to focus detection using the previously mentioned algorithm, and the results are shown in Figure 8. The algorithm indicated that the number of red pixels was at its lowest at 7.4 s, which corresponds with the data from the generated video.

**Figure 9:**
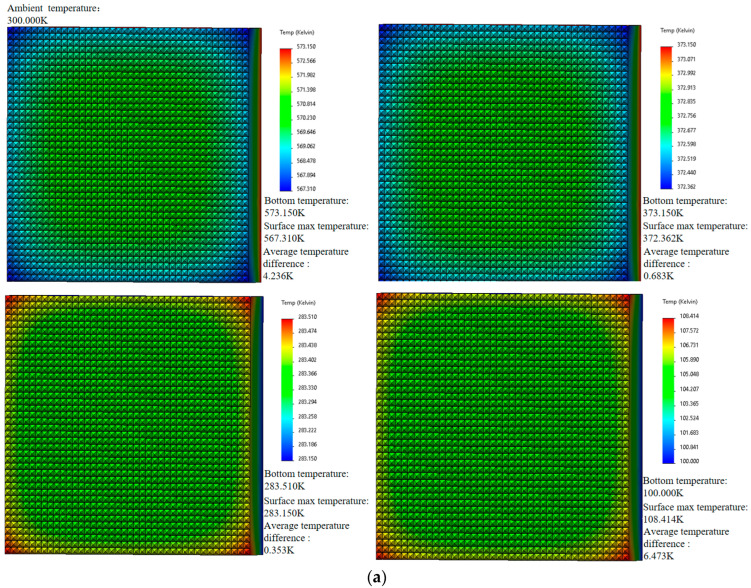

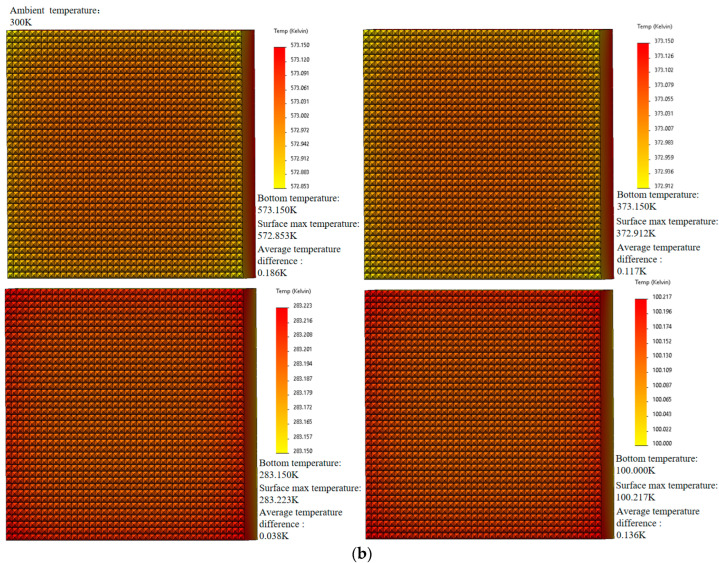
(**a**) The simulated surface temperature differences before correction at four distinct temperature points: 100 K, 283 K, 373 K, and 573 K. (**b**) The simulated surface temperature differences after correction at the same temperature points: 100 K, 283 K, 373 K, and 573 K.

The temperature data collected from a dual infrared thermometer at a single measurement point were input into a thermal model for simulation. Four temperature points commonly used for camera/telescope calibration—100 K, 283 K, 373 K, and 573 K—were selected for pre- and post-correction thermodynamic simulation and set the ambient temperature to 300 K. The results are presented in Figure 9. At a single blackbody surface temperature of 100 K, the maximum error decreased from 6.473 K before correction to 0.136 K after correction. At 573 K, the error reduced from 4.236 K to 0.186 K; at 373 K, the maximum error decreased from 0.683 K to 0.117 K; and at 283 K, the maximum error dropped from 0.353 K to 0.038 K.

## 4. Experimental Measurement

**Figure 10:**
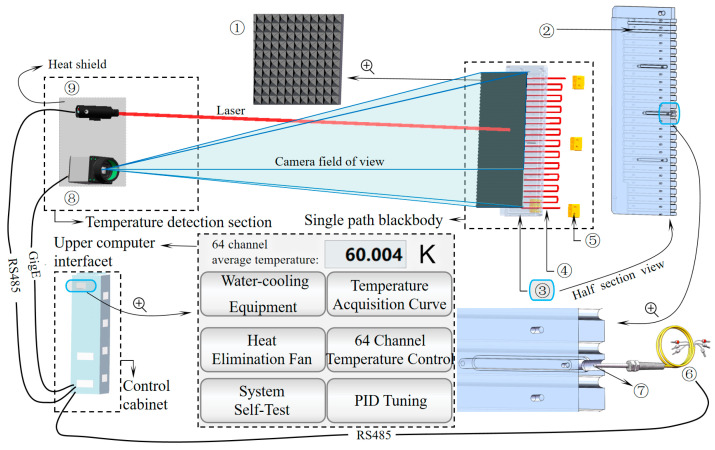
Schematic of the large-area blackbody temperature auto-correction system.

The schematic diagram of the large-scale blackbody temperature automatic correction system is shown in Figure 10. The focus adjustment platform was used to adjust the focus distance between the ⑨ dual infrared thermometer and the ① blackbody surface. The ⑧ camera continuously captured the focused laser spot of the infrared thermometer on the blackbody surface. The camera utilized the focus detection algorithm presented in Figure 3, integrated with the motor’s application of the optimal focus finding algorithm outlined in Figure 6, to identify the optimal measurement point. Subsequently, the temperature data from the infrared thermometer were transmitted to the host computer. The host computer compared the actual front surface temperature provided by the thermometer with the rear surface temperature measured by the ⑥ PT100 in the ⑦ PT1000 mounting hole on the ③ vapor chamber. An interpolated temperature value was obtained, which was transmitted to the temperature controller after weighing. The temperature controller then recalibrated the ④ heating wire in the ② heating wire installation slot behind the vapor chamber (fixed within the slot by ⑤ ceramic terminal blocks) to achieve a front surface temperature accuracy of 0.01 K.

**Figure 11:**
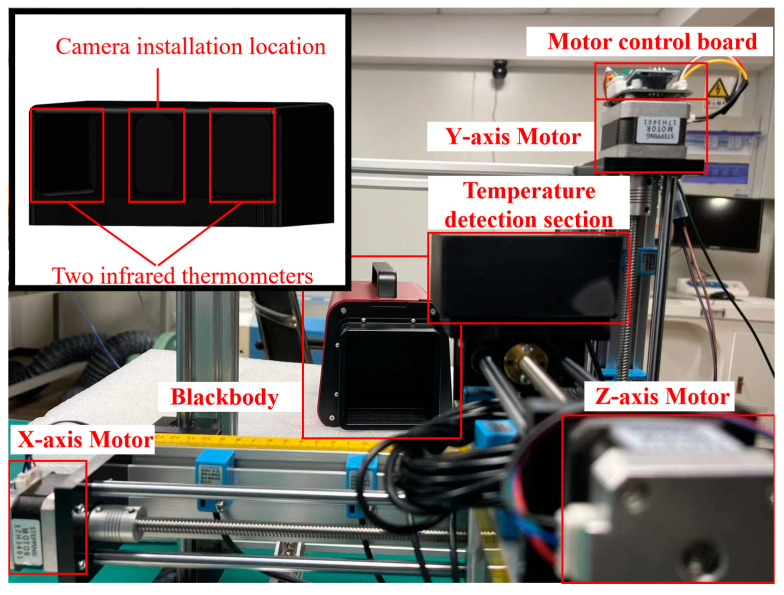
Small-scale verification platform physical image.

**Figure 12:**
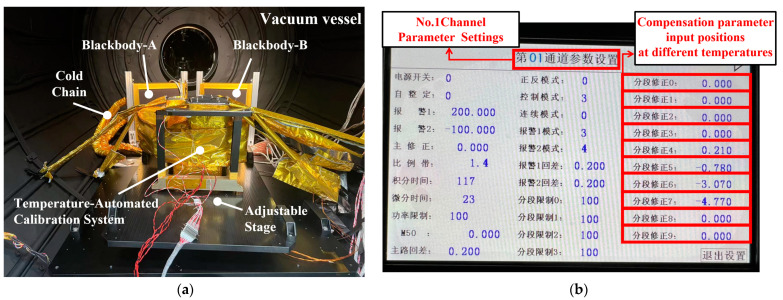
(**a**) Small-scale verification platform low-temperature test. (**b**) Temperature control channel parameter settings; the displayed temperature unit is °C.

The real object diagram of the small-scale verification platform is depicted in Figure 11. This platform was utilized to perform temperature correction on the surface of the small-scale blackbody, thereby validating the correction effect of the self-correcting platform. The low-temperature experimental testing platform, as shown in Figure 12a, was used to test the working conditions of the small-scale system in low-temperature environments after wrapping it with multi-layer insulation (MLI) materials made of polyimide and placing it into a vacuum canister. The blackbody parameters used in Figure 11 and Figure 12a are shown in Table 1. The adjustment platform was used to alter the direction of the automatic temperature correction system to achieve temperature correction for blackbody A and blackbody B. Due to the high time and economic costs associated with low-temperature testing, this experiment is conducted in conjunction with the lunar exploration camera experiment while also verifying the performance of the self-correction system. Blackbody A is used to simulate a constant temperature blackbody and a space environment background, while Blackbody B is used to simulate material on the moon. The compensation parameter input interface for different temperatures in a single temperature control channel is shown in Figure 12b. The weighted temperature compensation values obtained in experimental verification are inputted through this interface, and temperature control is performed again. The temperature value of the rear surface of the blackbody is obtained through PT1000, and the temperature value of the front surface is measured through our laboratory’s self-produced thermopile infrared sensor. For example, the temperature compensation value at 573 K is 3.07 K. After inputting this parameter, the rear surface will be heated by 3.07 K to compensate for the temperature difference between the front and rear surfaces, making the temperature of the black body front surface closer to 573 K.

**Figure 13:**
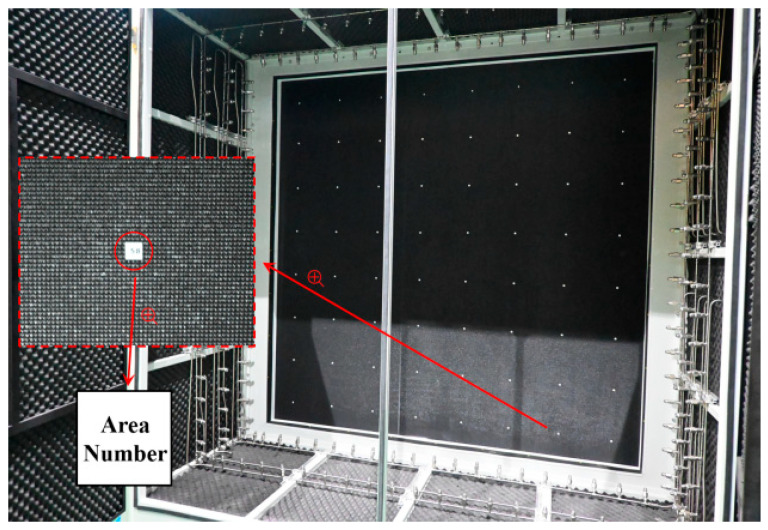
Large-area blackbody physical image.

**Figure 14:**
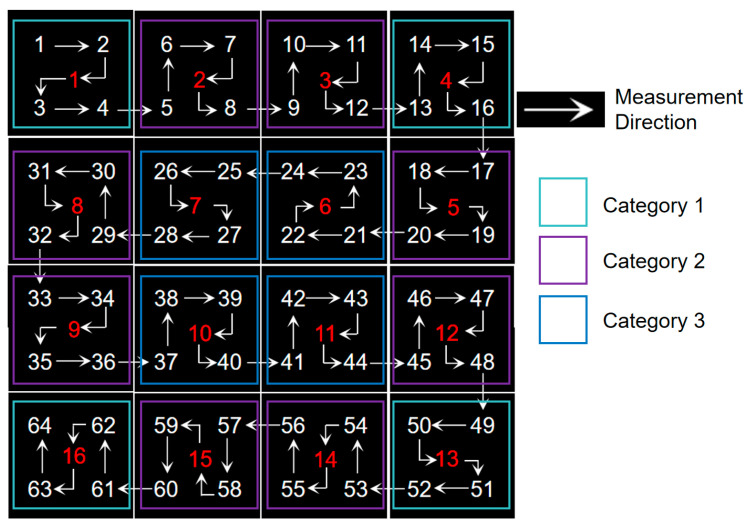
Sixteen-channel temperature measurement points and correction pathway.

The large-area blackbody auto-correction scheme involved using a large-area blackbody with dimensions of 2200 mm × 2200 mm and 64 temperature control channels, as depicted in Figure 13. The auto-correction system’s 64-channel temperature control correction pathway was similar to the 16-channel temperature correction scheme shown in Figure 14. Temperature measurements were taken at five points for each channel of the large-area blackbody: top left, top right, center, bottom left, and bottom right. The entire black square in Figure 14 represents a large-area blackbody radiation source; the red numbers represent the center of each temperature control channel; and the white numbers represent the four temperature measurement positions on the top left, top right, bottom left, and bottom right of each channel. Depending on the channel location, they were categorized into the three categories shown in Figure 14. The first category is enclosed by a light blue square frame in the diagram, which has three temperature measurement points in contact with the external environment. Assigned a weight of 15% to the three environmental contact points, 50% to the center point, and 5% to the remaining point. The weighted temperature value was then transmitted to the host computer for recalibration. The second category is enclosed by a purple square frame in the diagram, which has two temperature measurement points in contact with the external environment. Assigned a weight of 15% to the two environmental contact points, 50% to the center, and 10% to the other two points. The weighted temperature values were transmitted to the host computer for temperature compensation control. The third category is enclosed by a blue square frame in the diagram, and all five temperature measurement points in this category are not in contact with the external environment. Assign a weight of 5% to each of the four points and 80% to the center. The weighted temperature values were transmitted to the host computer for temperature compensation control.

## 5. Discussion

We have demonstrated through experiments that the auto-correction system has improved compared to mainstream manual correction in four aspects: temperature correction accuracy, large-area blackbody temperature uniformity, temperature measurement point consistency, and correction efficiency.

**Figure 15:**
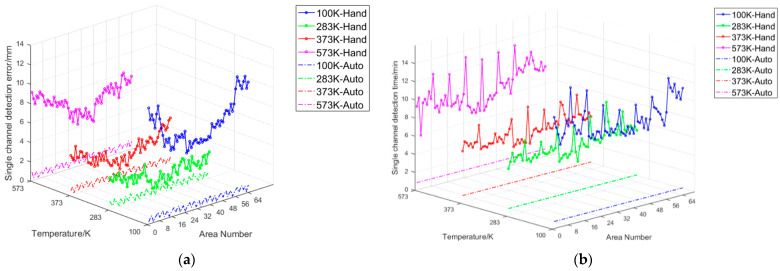
(**a**) Comparison of consistency errors between the manual and automatic calibration of single-channel temperature measurement points. (**b**) Comparison of the manual and automatic calibration single-channel temperature measurement times.

**Figure 16:**
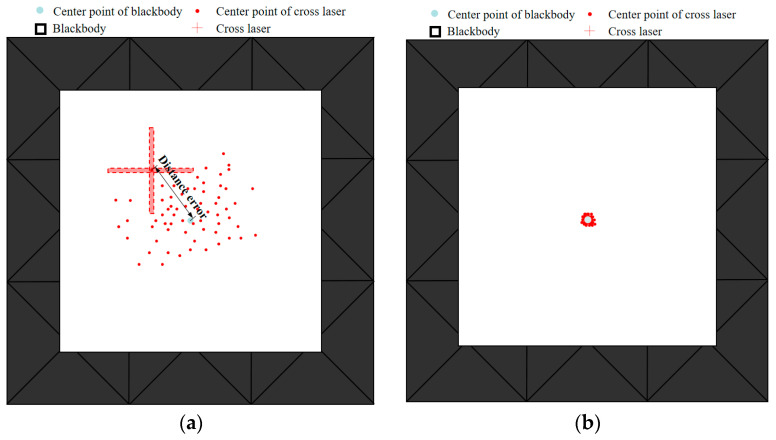
(**a**) Manual correction error distribution. (**b**) Automatic correction error distribution.

In the experimental validation, for the performance verification of auto-correcting systems at four different temperatures of 100 K, 283 K, 373 K, and 573 K, we used a low-temperature blackbody with a surface source size of 500 × 500 mm and 16 temperature-controlled channels for the 100 K experiment. We used a low-temperature blackbody composed of 64 temperature-controlled channels with a surface source size of 2200 × 2200 mm for experiments at 283 K and 373 K. A high-temperature blackbody with a surface source size of 1300 × 1300 mm and 32 temperature-controlled channels was used for the 573 K experiment. Under the temperature conditions of 283 K and 373 K, automatic and manual corrections were performed on five temperature measurement points for each of the 64 channels of the room temperature blackbody, as shown in Figure 14. Three repeated experiments were conducted under each temperature condition. Under the 100 K temperature condition, three repeated experiments were conducted on 80 temperature points of the low-temperature blackbody across 16 channels, with every four measurements as a group. Under the 573 K condition, three repeated experiments were conducted on 160 temperature points of the high-temperature blackbody across 32 channels, with every two measurements as a group. The mean distance error of each channel’s five temperature measurement points from the center point was calculated for the three types of blackbodies. The 64-channel temperature measurement point consistency error curve is shown in Figure 15a and the distribution of single-channel temperature measurement point consistency error data is shown in Figure 16. As seen in the figure, at 100 K, the average consistency error of automatic correction decreased from 8.69 mm upon manual correction to 0.57 mm, a reduction of 93.4%. At 283 K, it decreased from 2.16 mm to 0.52 mm, a reduction of 75.9%. At 373 K, it decreased from 2.93 mm to 0.56 mm, a reduction of 80.9%. Finally, at 573 K, it decreased from 6.68 mm to 0.57 mm, a reduction of 91.5%. The average consistency error decreased by 85.4% under the four temperature conditions.

The comparison of single-channel correction times is shown in Figure 15b. As can be seen from the figure, at 100 K, the average correction time of automatic correction for each channel was reduced from 10.15 min upon manual correction to 0.81 min, an increase in correction efficiency of 12.5 times. At 283 K, it was reduced from 6.05 min to 0.82 min, an increase of 7.38 times. At 373 K, it was reduced from 6.09 min to 0.78 min, an increase of 7.81 times. At 573 K, it was reduced from 9.28 min to 0.80 min, an increase of 11.6 times. The correction efficiency increased by an average of 9.82 times under the four temperature conditions.

The consistency of the temperature measurement points plays a decisive role in judging the uniformity index of large-area blackbody temperature. After three manual and automatic corrections, the resulting comparison of blackbody surface temperature accuracy is shown in Table 2. At 100 K, the temperature measurement deviation of automatic correction decreased from 0.781 K upon manual correction to 0.456 K, a reduction of 41.6%. At 283 K, it decreased from 0.011 K to 0.007 K, a reduction of 36.4%. At 373 K, it decreased from 0.035 K to 0.017 K, a reduction of 51.4%. At 573 K, it decreased from 1.023 K to 0.693 K, a reduction of 32.3%. The average temperature measurement deviation decreased by 40.4% under the four temperature conditions.

The comparison of temperature uniformity is shown in Table 3. At 100 K, the surface temperature uniformity of automatic correction decreased from 0.437 K in manual correction to 0.283 K, a reduction of 35.2%. At 283 K, it decreased from 0.116 K to 0.071 K, a reduction of 38.7%. At 373 K, it decreased from 0.725 K to 0.327 K, a reduction of 54.9%. At 573 K, it decreased from 2.213 K to 1.189 K, a reduction of 46.3%. The average temperature uniformity increased by 43.8% under the four temperature conditions.

The bottleneck of this correction method is to find a temperature measurement point weight distribution that is suitable for all heating device arrangements. At present, the weights used in this system are only applicable to the heating device layout used in our laboratory, and the effect is significant. No experiments have been conducted on the correction effect of other layout methods. However, since the correction principle is to replace manual correction with automatic correction, it should also have advantages in other heating device arrangements, except that the distribution of the weight proportion of temperature measurement points will affect how much performance can be improved.

## 6. Conclusions

This paper proposes a method for the automatic correction of large-area blackbody surface temperatures within the temperature range of 100–573 K. Before the start of this work, the field of temperature correction for large-area blackbody radiation sources mainly relied on manual correction, and this work was an innovative proposal. Compared to traditional manual correction, it has better correction efficiency, temperature correction accuracy, and surface source temperature uniformity, as shown in Table 4. We conducted experimental tests comparing automatic and manual corrections on the same blackbody produced in our laboratory, evaluating the consistency of temperature measurement points, correction efficiency, temperature measurement deviation, and temperature uniformity after three correction operations. The results indicate that, compared to manual correction, automatic correction reduces the average error in temperature measurement point consistency by 85.4%, increases correction efficiency by an average of 9.82 times, reduces temperature measurement deviation by an average of 40.4%, and enhances temperature uniformity by an average of 43.8%. These findings underscore the practical value of the proposed automatic correction method for blackbody surface temperatures in the manufacturing of blackbodies.

## Figures and Tables

**Table 1:** The utilized parameters of the blackbody in the small-scale model validation experiment conducted within a vacuum chamber.

Blackbody emitter size	500 mm × 500 mm
Operating temperature range	50 K–360 K
Temperature resolution	0.001 K
Temperature accuracy	0.01 K
Effective emissivity	0.99
Temperature uniformity	≤0.5 K
Temperature control stability	0.02 K/30 min

**Table 2:** Comparison of the temperature measurement error of the same blackbody developed by our laboratory under automatic and manual calibration.

Temperature/K	The Average Difference between the Blackbody Surface Source Temperature and the Actual Temperature T_TA_/K		The Size of the Blackbody/mm	Average Ambient Temperature/K	Number of Temperature Controled Channels
	Manual	Automatic			
100	0.781	0.456	500 × 500	80.278	16
283	0.011	0.007	2200 × 2200	301.872	64
373	0.035	0.017	2200 × 2200	301.981	64
573	1.023	0.693	1300 × 1300	301.102	32

**Table 3:** Comparison of temperature uniformity of the same blackbody developed by our laboratory under automatic and manual calibration.

Temperature/K	Corrected Blackbody Surface Source Actual Temperature Uniformity $∆T_{F}$/K		The Size of the Blackbody/mm	Average Ambient Temperature/K	Number of Temperature Controled Channels
	Manual	Automatic			
100	0.437	0.283	500 × 500	80.273	16
283	0.116	0.071	2200 × 2200	301.872	64
373	0.725	0.327	2200 × 2200	301.981	64
573	2.213	1.189	1300 × 1300	301.102	32

**Table 4:** The average performance improvement in temperature-automated calibration methods for a large-area blackbody radiation source compared to traditional manual correction.

Performance Name	Increase the Proportion
Temperature measurement point consistency	85.4%
Temperature measurement deviation	40.4%
Temperature uniformity	43.8%
Correction efficiency	9.82 Times

## Data Availability

Data are contained within the article.
